# Incidence and risk factors for postoperative pulmonary complications in children following surgery for retroperitoneal neuroblastoma

**DOI:** 10.3389/fped.2026.1817205

**Published:** 2026-05-19

**Authors:** Xinran He, Jing Hu, Jianmin Zhang, Fang Wang

**Affiliations:** Department of Anesthesiology, Beijing Children’s Hospital, Capital Medical University, National Center for Children’s Health, Beijing, China

**Keywords:** children, neuroblastoma, postoperative pulmonary complications, prediction model, risk factors

## Abstract

**Introduction:**

Neuroblastoma is a common extracranial solid tumor in children, associated with high perioperative risk. Postoperative pulmonary complications, such as pneumonia and pleural effusion, occur frequently and may adversely affect patient prognosis. However, the underlying risk factors remain incompletely understood. This study aimed to investigate risk factors for pulmonary complications following neuroblastoma resection and to establish predictive cutoff values for clinical risk stratification.

**Methods:**

This single-center retrospective study included 214 children who underwent elective abdominal neuroblastoma resection between April 2018 and December 2020. The primary endpoint was the incidence of postoperative pulmonary complications. Preoperative, intraoperative, and postoperative variables were analyzed using univariate and multivariate logistic regression. Receiver operating characteristic analysis was performed to determine cutoff values for clinical parameters.

**Results:**

The incidence of postoperative pulmonary complications was 26.2%. Logistic regression analysis identified three independent risk factors: postoperative day 1 albumin concentration (cutoff: 32.9 g·L⁻^1^; adjusted odds ratio [OR] 1.268; 95% confidence interval [CI] 1.086–1.482; *P* = 0.003), preoperative C-reactive protein (CRP) concentration (cutoff: 9.5 mg·L⁻^1^; adjusted OR 1.357; 95% CI 1.117–1.648; *P* = 0.002), and postoperative day 1 CRP concentration (cutoff: 19.5 mg·L⁻^1^; adjusted OR 1.037; 95% CI 1.003–1.072; *P* = 0.032).

**Conclusion:**

This study identifies critical thresholds for identifying children at high risk of pulmonary complications after neuroblastoma resection, facilitating more accurate risk stratification and early intervention. These findings have important clinical implications for optimizing perioperative management and reducing postoperative morbidity.

## 1 Introduction

Neuroblastoma is one of the most common extracranial solid tumors in children, with an incidence ranging from 0.5 to 1.5 per 100,000 population (1). Surgical resection serves as the cornerstone of neuroblastoma treatment and can significantly improve overall prognosis, yet postoperative complications often adversely affect recovery and long-term outcomes. The incidence of postoperative pulmonary complications (PPCs) in children ranges from 3.1% to 25.9% (2–5), with common manifestations including pneumonia, atelectasis, pleural effusion, and hypoxemia. Early interventions such as lung-protective ventilation strategies can effectively alleviate PPC-related symptoms (6). Children are particularly susceptible to PPCs due to their unique respiratory physiology (7, 8), while systematic research on neuroblastoma-specific risk and protective factors for PPCs remains insufficient (9). This study aims to comprehensively analyze potential risk factors by collecting and evaluating perioperative medical records of neuroblastoma patients, with the goal of precisely identifying independent risk factors and potential protective factors while assessing their predictive value. The findings will provide critical evidence to reduce PPC incidence, develop individualized prevention strategies, alleviate hospitalization burdens, optimize postoperative recovery, and ultimately improve treatment protocols for children with neuroblastoma.

## 2 Methods

This study was approved by the Ethics Committee of Beijing Children's Hospital, Capital Medical University [Ethical Approval No.: (2025)-E-199-R], and has been registered with the Chinese Clinical Trial Registry (Registration No.: ChiCTR2600116850). Medical records of children who underwent retroperitoneal neuroblastoma resection at our hospital from April 2018 to December 2020 were retrospectively collected. Inclusion criteria were: ① underwent retroperitoneal neuroblastoma resection during the study period; ② age <18 years. Exclusion criteria included: ① Preoperative presence of lung disease; ② Prematurity; ③ Congenital heart disease or other severe cardiac conditions; ④ Intraoperative diaphragmatic or pancreatic injury; ⑤ Incomplete laboratory test results; ⑥ Postoperative transfer to the ICU or requirement for mechanical ventilation.

Relevant medical record data were retrieved and collected through the Jiahe Electronic Medical Record System and the DoCare Anesthesia Information System, including: ① General patient information: age, sex, height, weight, ethnicity, ASA physical status classification; ② Laboratory tests: hemoglobin, C-reactive protein (CRP), creatinine, and albumin levels within 7 days preoperatively and within 3 days postoperatively; ③ Tumor-related information: International Neuroblastoma Risk Group (INRG) risk category and stage, tumor location, maximum diameter, number of lymph node dissection areas, and pathological type; ④ History of preoperative chemotherapy; ⑤ Intraoperative details: operative time, total fluid intake, use of blood products, estimated blood loss, urine output, arterial blood gas analysis results, number of lymph node dissection areas; ⑥ Chest imaging findings and occurrence of thoracentesis within 7 days postoperatively.

The incidence of postoperative pulmonary complications (PPCs) was calculated based on the diagnostic criteria for PPCs established by the American Society of Anesthesiologists (ASA), as defined in Table 1 (10). Patients were divided into a PPCs group and a non-PPCs group based on the occurrence of PPCs within 7 days after surgery.

**Table 1:** Definitions of postoperative pulmonary complications.

Complication	Definition
Respiratory infection	When a patient received antibiotics for suspected respiratory infection and met at least one criterion including new or changed sputum, new or changed lung opacities, fever, or leukocyte count >12,000/μL
Respiratory failure	When postoperative PaO_2_ < 60 mmHg on room air, a PaO_2_/inspired oxygen fraction ratio <300, or pulse oximetry <90% requiring oxygen therapy
Pleural effusion	Chest x-ray findings such as blunting of the costophrenic angle, loss of the ipsilateral hemidiaphragm silhouette, displacement of adjacent structures, or hazy opacity with preserved vascular shadows
Atelectasis	Lung opacification with mediastinal, hilar, or diaphragmatic shift toward the affected area and compensatory overinflation
Pneumothorax	Air in the pleural space without surrounding vascular bed
Bronchospasm	Newly detected expiratory wheezing treated with bronchodilators
Aspiration pneumonitis	Acute lung injury following inhalation of regurgitated gastric contents

Statistical analysis was performed using SPSS software (version 26.0). Normally distributed quantitative data are presented as mean ± standard deviation (*X* ± s) and compared between groups using the independent samples *t*-test. Non-normally distributed measurement data are presented as median (interquartile range) [*M* (IQR)] and compared using the Mann–Whitney *U* test. Variables with *P* < 0.05 in univariate analyses were included in a multivariate logistic regression model to identify independent risk factors for PPCs, the specific method used for multivariate logistic regression was enter method. Receiver operating characteristic (ROC) curves were plotted to evaluate the predictive performance of the risk factors. The Youden index was calculated, with the maximum Youden index value determining the optimal cutoff point. A *P*-value <0.05 was considered statistically significant.

## 3 Results

**Figure 1:**
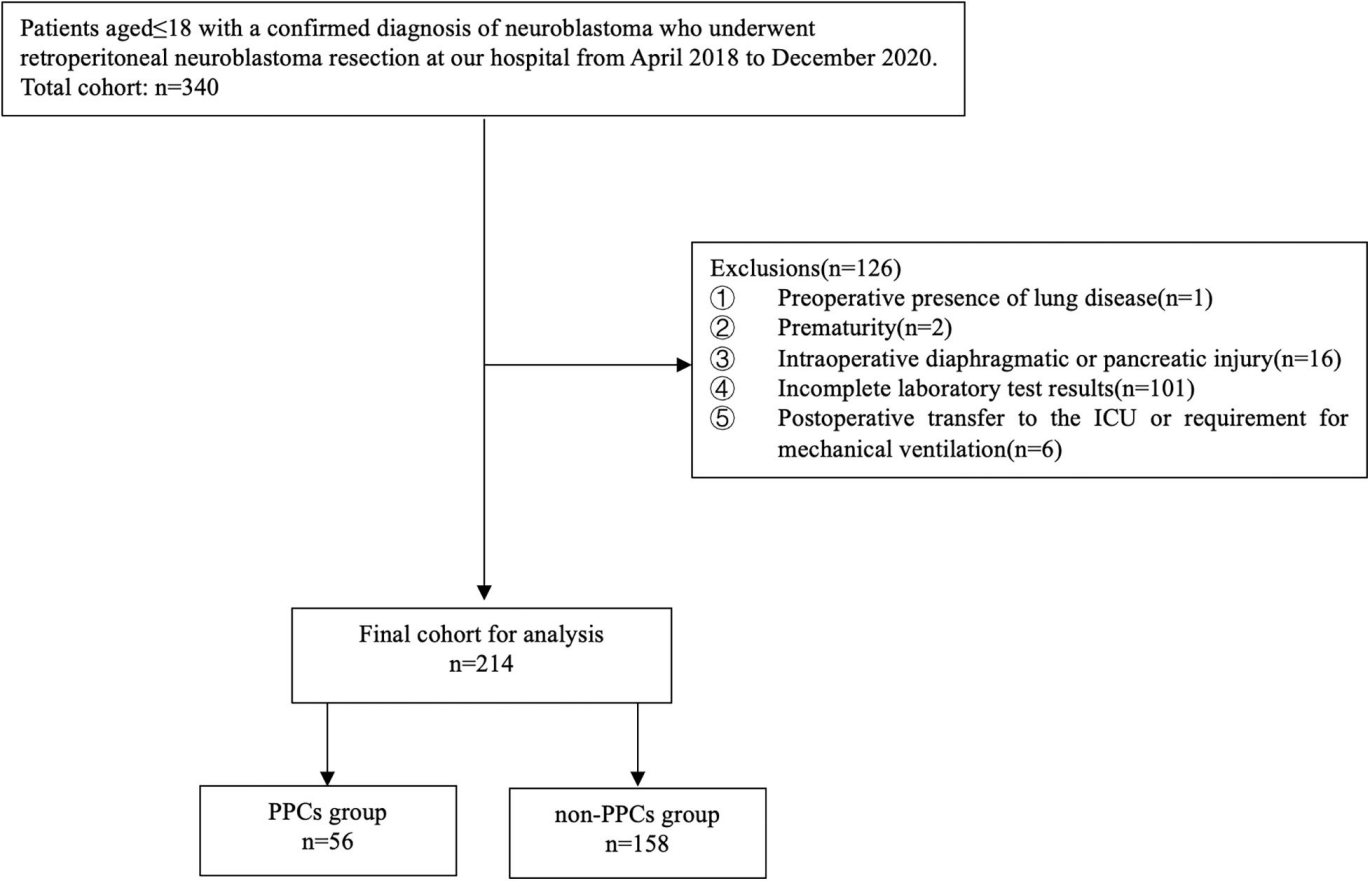
Study enrollment flowchart.

A total of 340 patients aged 18 and below underwent resection of retroperitoneal neuroblastoma. After excluding cases that did not meet the inclusion criteria, 214 patients were finally included for analysis (see Figure 1). Among them, 56 developed PPCs within 7 days postoperatively, corresponding to an incidence of 26.2%. Pleural effusion (*n* = 41) and pneumonia (*n* = 39) were the only complications observed: 29 patients presented with pleural effusion alone, 27 with pneumonia alone, and 12 with both. A representative chest x-ray of a patient with PPCs is shown in Supplementary Figure S1. No cases of respiratory failure, atelectasis, pneumothorax, bronchospasm, or aspiration pneumonia were documented. Based on the occurrence of PPCs, patients were divided into two groups: the PPCs group (*n* = 56) and the non-PPCs group (*n* = 158). Demographic, clinical, and surgery-related baseline characteristics are shown in Table 2. There were no statistically significant differences between the two groups in terms of age, sex, ethnicity, height, weight, ASA classification, or tumor location (*P* > 0.05).

**Table 2:** Study group characteristics and univariate analysis results for postoperative pulmonary complications.

Variables	Overall, *n* = 214^a^	Postoperative pulmonary complications		*P*-value*
		Yes (*n* = 56)	No (*n* = 158)	
		*n* = 56 (26.2%)^a^	*n* = 158 (73.8%)^a^	
Demographics				
Sex				0.343
Girl	103 (48.1%)	30 (29.1%)	73 (70.9%)	
Boy	111 (51.9%)	26 (23.4%)	85 (76.6%)	
Age, month	37.0 [19.0–59.0]	37.0 [18.0–60.0]	37.0 [19.3–57.8]	0.677
Ethnicity				0.609
Han	191 (89.3%)	51 (91.1%)	140 (88.6%)	
Minority	23 (10.7%)	5 (8.9%)	18 (11.4%)	
Preoperative status				
Paediatric ASA classification				0.057
III	27 (12.6%)	3 (5.4%)	24 (15.2%)	
Ⅰ–II	187 (87.4%)	53 (94.6%)	134 (84.8%)	
BMI	16.5 [14.9–18.4]	15.8 [14.7–17.0]	16.6 [15.0–18.8]	0.021
Preoperative chemotherapy history				<0.001
Yes	135 (63.1%)	47 (83.9%)	88 (55.7%)	
No	79 (36.9%)	9 (16.1%)	70 (44.3%)	
Preoperative C-reactive protein, mg.l-1	5.0 [5.0–8.0]	8.0 [5.0–15.0]	5.0 [5.0–6.8]	<0.001
Preoperative haemoglobin, g.L^−1^	107 [96.0–121.0]	101 [90.0–111.0]	109 [97.0–124.0]	0.001
Preoperative albumin, g.L^−1^	42.3 [40.0–44.7]	42.0 [38.6–45.0]	42.4 [40.0–44.7]	0.338
Preoperative creatinine, μmol.L^−1^	23.4 [20.0–29.0]	21.0 [18.1–27.0]	24.0 [20.4–30.1]	0.003
Tumour characteristics				
Pathology				0.026
NB	122 (57.0%)	39 (69.6%）	83 (52.5%)	
Others (ganglioneuroma and ganglioneuroblastoma)	92 (43.0%)	17 (30.4%)	75 (47.5%)	
International neuroblastoma risk group stage				<0.001
M	99 (46.3%)	40 (71.4%)	59 (37.3%)	
Others	115 (53.7%)	16 (28.6%)	99 (62.7%)	
International neuroblastoma risk group risk stratification				<0.001
High risk	113 (52.8%)	41 (73.2%)	72 (45.6%)	
Others	101 (47.2%)	15 (26.8%)	86 (54.4%)	
Tumour origin				0.782
Adrenal	142 (66.4%)	38 (67.9%)	104 (65.8%)	
Retroperitoneum	72 (33.6%)	18 (32.1%)	54 (34.2%)	
Surgical procedure				
Operative time, hour	4.2 [3.0–6.0]	6.0 [4.7–8.0]	3.5 [2.8–5.0]	<0.001
Number of resected lymphatic regions	3.0 [1.0–4.0]	5.0 [3.0–7.0]	2.0 [1.0–4.0]	<0.001
Maximum diameter, cm	6.0 [4.0–8.1]	7.3 [5.0–10.0]	5.6 [3.6–8.0]	0.005
Perioperative fluid management				
Estimated blood loss, mL.kg^−1^	10.0 [7.0–30.0]	30.0 [20.0–50.0]	10.0 [5.0–20.0]	<0.001
Volume of infused crystalloids, mL.kg^−1^.h^−1^	12.5 ± 4.6	14.6 ± 4.0	11.7 ± 4.5	<0.001
Volume of urine output, mL.kg^−1^.h^−1^	2.1 [1.4–3.2]	2.1 [1.5–3.1]	2.1 [1.4–3.3]	0.972
Postoperative day 0 (day of surgery)				
C-reactive protein, mg.L^−1^	14.0 [6.0–26.0]	23.0 [12.0–49.0]	11.0 [5.0–22.8]	<0.001
Albumin, g.L^−1^	35.1 [32.3–38.6]	31.1 [26.6–33.7]	36.5 [34.0–39.0]	<0.001
Creatinine, μmol.L^−1^	22.3 [18.0–28.4]	21.0 [16.9–28.4]	23.2 [18.4–28.6]	0.363
Haemoglobin, g.L^−1^	100 [89.0–112.0]	99.0 [87.0–110.0]	101.5 [90.3–113.0]	0.386
Impact of PPCs on postoperative management and mortality				
Cost, Pound	4,137 [3,379–5,111]	6,064 [4,751–7,684]	3,714 [3,227–4,496]	<0.001
Mortality	29 (13.6%)	8 (14.3%）	21 (13.4%)	0.865

a Median [IQR]; *n* (%) or mean ± sd; *n*(%).

* Wilcoxon rank sum test; Pearson's Chi-squared test; Fisher' Exact Test for Count Data with simulated *P*-value.

Univariate logistic regression analysis showed that BMI, perioperative laboratory indicators (preoperative hemoglobin, CRP, creatinine levels within 7 days; postoperative CRP and albumin levels within 3 days), tumor characteristics (INRG risk category and stage, maximum diameter, number of lymph node dissection areas, pathological type), operative time, intraoperative fluid intake per body weight, estimated blood loss, and history of preoperative chemotherapy were significantly associated with the occurrence of postoperative PPCs. To identify independent risk factors, variables with *P* < 0.05 in the univariate analysis were included in a multivariate logistic regression model (see Table 3).

**Table 3:** Results of multivariate logistic regression analysis for predictive factors of postoperative pulmonary complications.

Variables	*P*-value^*^	aOR^a^	(95% CI)^a^	
			Lower	Upper
BMI	0.230	0.830	0.612	1.125
Operative time	0.095	1.312	0.954	1.805
Maximum diameter of tumour at surgery	0.916	0.988	0.793	1.231
Number of resected lymphatic regions	0.201	1.191	0.911	1.558
Volume of infused crystalloids	0.469	1.058	0.908	1.232
Estimated blood loss	0.085	1.012	0.998	1.026
Preoperative haemoglobin	0.787	0.993	0.944	1.045
Preoperative C-reactive protein	0.002	1.357	1.117	1.648
Preoperative creatinine	0.987	0.999	0.866	1.152
C-reactive protein on postoperative day	0.032	1.037	1.003	1.072
Albumin on postoperative day	0.003	1.268	1.086	1.482
Pathology	0.419	1.693	0.472	6.073
International neuroblastoma risk group stage	0.058	0.178	0.030	1.061
International neuroblastoma risk group risk stratification	0.090	5.045	0.775	32.829
Preoperative chemotherapy history	0.322	2.960	0.345	25.390

Full regression details are provided in Supplementary Table S1.

a Only odds ratios (OR), 95% CI, and *P-values are shown.

**Figure 2:**
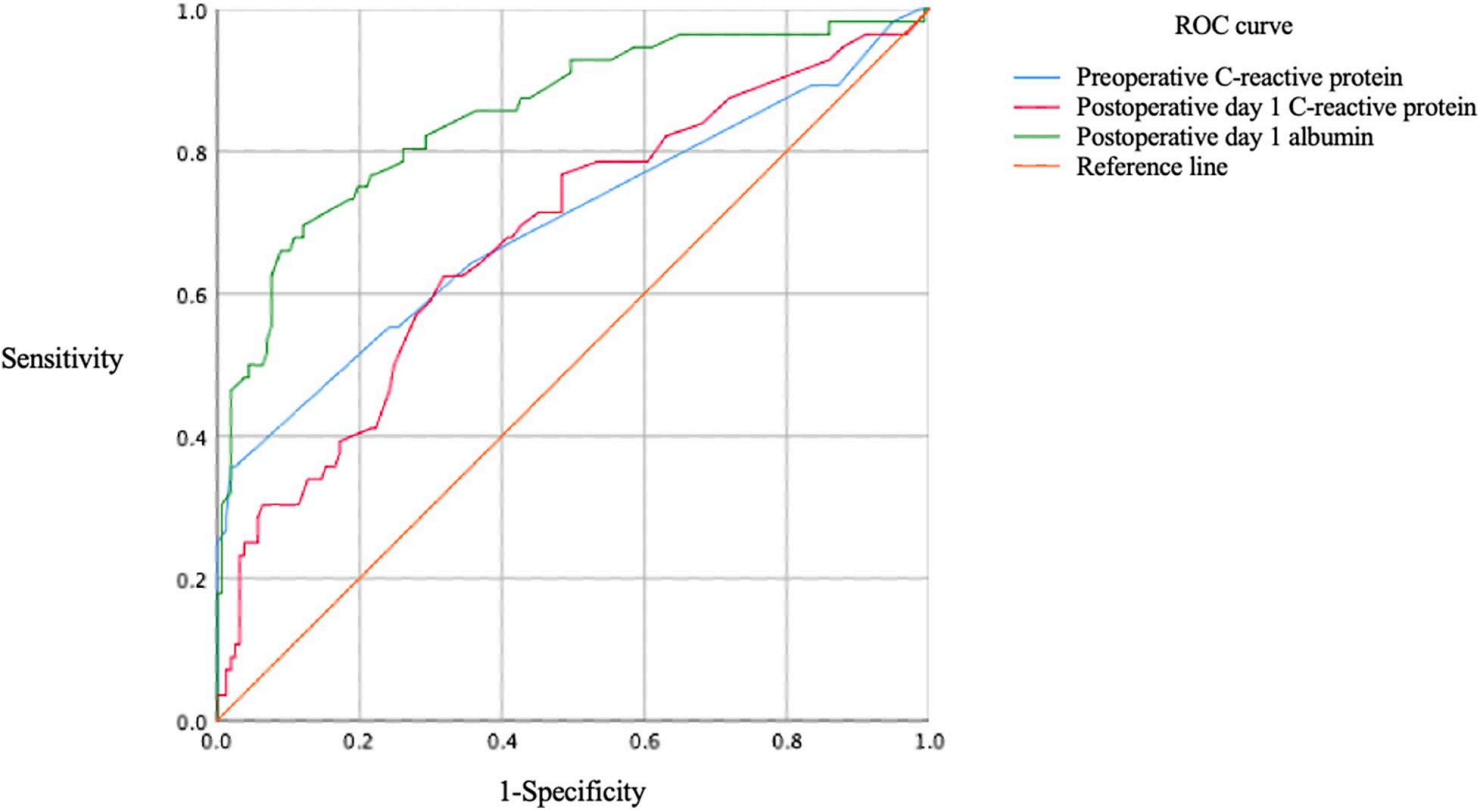
ROC curve of independent risk factors.

**Figure 3:**
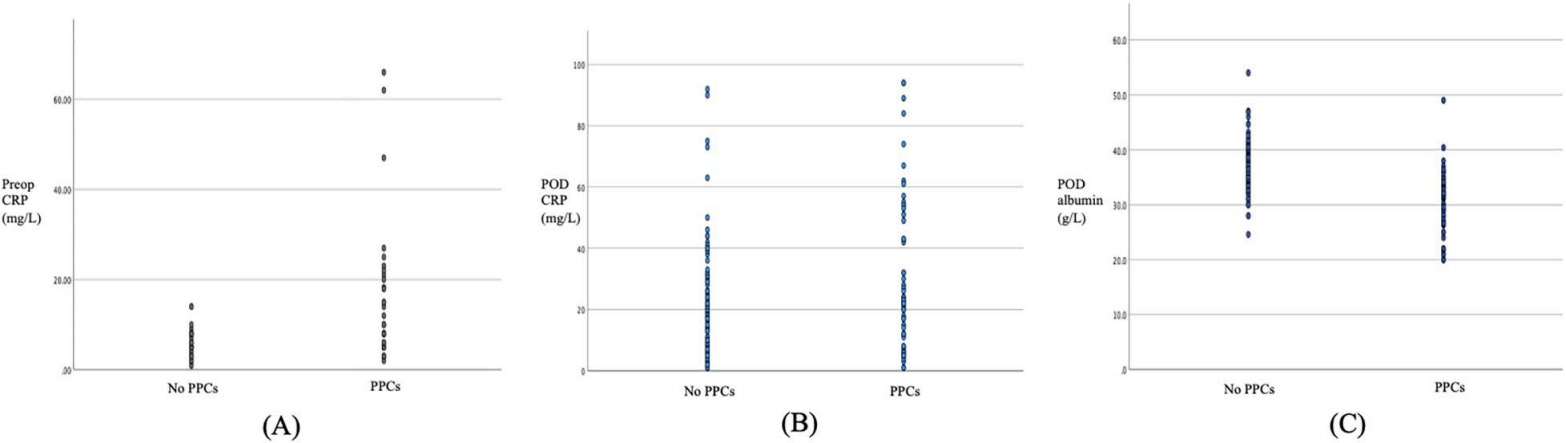
Scatter plots showing the distributions of the three independent risk factors between patients with and without postoperative pulmonary complications (PPCs). **(A)** Preoperative CRP; **(B)** CRP on postoperative day; **(C)** albumin on postoperative day.

After adjusting for potential confounding factors, three laboratory indicators were identified as independent risk factors for PPCs, and the ROC curve is shown in Figure 2, the scatter plot is shown in Figure 3:

1. Preoperative C-reactive protein level: Elevated preoperative CRP was an independent risk factor for PPCs (adjusted odds ratio [aOR] = 1.357; 95% confidence interval [CI], 1.117–1.648; *P* = 0.002). ROC curve analysis showed an area under the curve (AUC) of 0.697 (95% CI, 0.607–0.787). Combined with the Youden index, the optimal cutoff value was determined to be 9.5 mg/L, with a sensitivity of 35.7% and specificity of 98.1%.
2. Postoperative day 1 C-reactive protein level: Elevated CRP on postoperative day 1 was an independent risk factor for PPCs (aOR = 1.037; 95% CI, 1.003–1.072; *P* = 0.032). ROC analysis yielded an area under the curve (AUC) of 0.680 (95% CI, 0.595–0.764). The optimal cutoff value was determined to be 19.5 mg/L, with a sensitivity of 62.5% and specificity of 68.2%.
3. Postoperative day 1 albumin level: Decreased albumin on postoperative day 1 was an independent risk factor for PPCs (aOR = 1.268; 95% CI, 1.086–1.482; *P* = 0.003). ROC analysis yielded an area under the curve (AUC) of 0.852 (95% CI, 0.789–0.915). The optimal cutoff value was 32.9 g/L, with a sensitivity of 69.6% and specificity of 87.9%.

**Figure 4:**
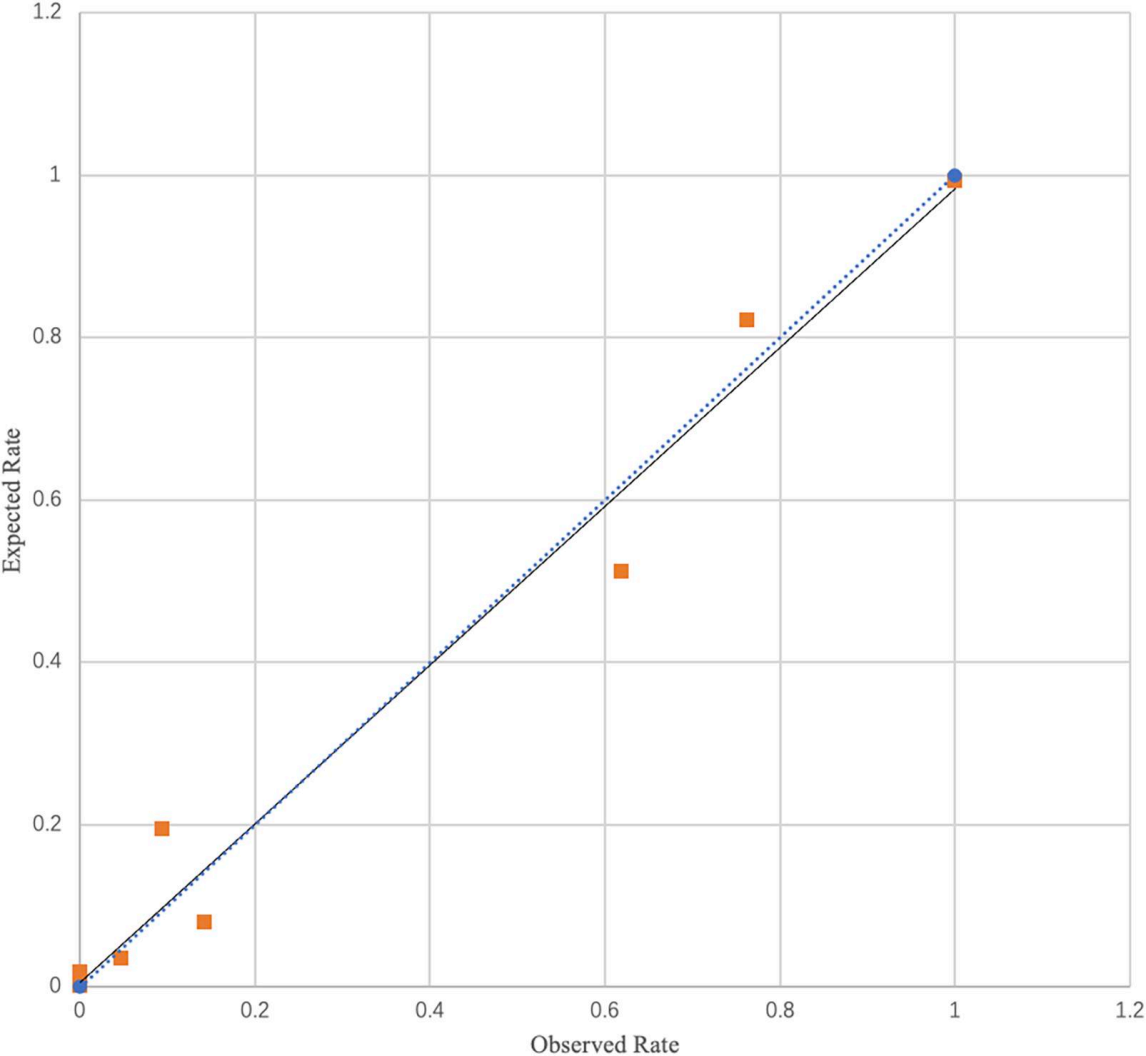
Calibration curve of Hosmer–Lemeshow test.

The prediction model constructed in this study demonstrated good overall performance. In terms of discrimination, the model's C-statistic (which is equivalent to the area under the ROC curve, AUC) was 0.956 (95% CI, 0.929–0.983), indicating excellent discriminatory ability. Calibration was assessed using the Hosmer–Lemeshow test (Figure 4), which showed no significant difference between the model-predicted risk and the observed risk (*χ*^2^ = 4.868, *P* = 0.772), suggesting good model calibration. Furthermore, the model's goodness-of-fit was quantified by the Nagelkerke *R*^2^ value of 0.739, indicating that the included predictors collectively explained approximately 74% of the variance in the outcome variable. The performance metrics of the three independent risk factors and the prediction model sees Table 4.

**Table 4:** Performance metrics of individual risk factors and the combined prediction model for postoperative pulmonary complications.

Predictor	AUC (95% CI)	Cutoff value	Sensitivity (%)	Specificity (%)	Accuracy (%)	PPV (%)	NPV (%)
Preoperative CRP (mg/L)	0.697 (0.607–0.787)	9.5	35.7	98.1	81.8	87.0	81.2
CRP on postoperative day (mg/L)	0.680 (0.595–0.764)	19.5	62.5	68.2	66.7	41.2	83.6
Albumin on postoperative day (g/L)	0.852 (0.789–0.915)	32.9	69.6	87.9	83.2	67.2	89.1
Combined prediction model^a^	0.956 (0.930–0.982)	0.375	81.8	94.8	91.4	84.9	93.6

AUC, area under the curve; CI, confidence interval; PPV, positive predictive value; NPV, negative predictive value.

a The combined prediction model includes preoperative CRP, CRP on postoperative day, and albumin on postoperative day.

In summary, this model reliably distinguished between high-risk and low-risk patients, and its predicted risk probabilities aligned well with the actual observed outcomes.

## 4 Discussion

Through a retrospective analysis of data from 214 pediatric patients undergoing neuroblastoma resection, this study systematically evaluated risk factors for postoperative pulmonary complications (PPCs). We found that the incidence of PPCs within 7 days postoperatively was 26.2%. After adjusting for confounders, elevated preoperative and postoperative day 1 C-reactive protein (CRP) levels, along with decreased postoperative day 1 albumin levels, were identified as three independent risk factors for PPCs. A prediction model integrating these indicators demonstrated excellent discrimination (AUC = 0.956) and calibration.

The relatively high PPC incidence rate (26.2%) in our study is consistent with previous reports on major pediatric surgeries, particularly abdominal tumor resections (7, 11), highlighting the necessity for precise perioperative risk assessment in this high-risk population. Pleural effusion and pneumonia were the most common PPC types, likely attributable to the extensive surgical field, prolonged operative time, and surgical manipulation near the thoracic cavity, abdomen, and diaphragm.

The perioperative physiology of children with abdominal neuroblastoma has distinct characteristics that provide a direct pathophysiological rationale for routine monitoring of inflammatory markers before and after surgery. First, neuroblastoma itself can induce a systemic tumor-associated inflammatory response, reflected by elevated levels of C-reactive protein (CRP) and other inflammatory markers. This chronic, low-grade inflammation may impair immune regulation and compromise pulmonary tissue repair following surgical trauma. Second, neuroblastomas often secrete catecholamines, and their mass effect can elevate the diaphragm and reduce chest wall compliance, placing these children in a state of limited respiratory reserve even before surgery (12). Together, these unique physiological features render children with abdominal neuroblastoma more susceptible to surgical trauma and perioperative inflammatory insults than children undergoing general abdominal surgery. Therefore, dynamic monitoring of markers reflecting systemic inflammatory burden (such as CRP) before and early after surgery holds particular clinical value for predicting the risk of postoperative pulmonary complications (PPCs).

The most significant finding of this study is the identification of the independent predictive value of CRP at two time points: preoperatively and early postoperatively. Elevated preoperative CRP (optimal cutoff 9.5 mg/L) may reflect a preexisting subclinical inflammatory state or tumor-associated systemic inflammation (13, 14). This chronic, low-grade inflammation could impair immune regulation and weaken pulmonary tissue repair following surgical trauma, thereby establishing a pathophysiological basis for PPCs. The sharp rise in CRP on postoperative day 1 (optimal cutoff 19.5 mg/L) directly reflects the magnitude of surgical trauma and the systemic inflammatory response (15, 16). Our results indicate that not only the preoperative inflammatory “baseline” is important, but also the postoperative inflammatory “peak” is critical for determining clinical outcomes. This suggests that dynamic perioperative monitoring of CRP, rather than a single measurement, is more meaningful for risk stratification.

Notably, decreased albumin on postoperative day 1 (aOR = 1.268) was also identified as an independent risk factor. Early postoperative hypoalbuminemia is a composite marker of malnutrition, inflammation-induced capillary leakage, and suppressed hepatic synthetic function (17–19). Low albumin reduces plasma colloid osmotic pressure, potentially leading to pulmonary interstitial edema, and may also affect immune function and drug binding, thereby increasing PPC risk through multiple pathways.

The multifactorial prediction model developed in this study demonstrated good discriminatory ability (AUC > 0.9) and excellent calibration (Hosmer–Lemeshow test, *P* = 0.772), indicating that combined assessment of inflammatory markers (CRP) and nutritional/synthetic function (albumin) can effectively identify children at high risk for postoperative pulmonary complications (PPCs). To enhance clinical applicability, we performed a simplified risk stratification using the three independent cutoff values: preoperative CRP ≥ 9.5 mg/L, postoperative day 1 CRP ≥ 19.5 mg/L, and postoperative day 1 albumin ≤32.9 g/L. Patients meeting at least two of these criteria were classified as high-risk. In our cohort of 214 patients, 44 (20.6%) were high-risk and 170 (79.4%) low-risk. The observed PPC incidence was 77.3% (34/44) in the high-risk group vs. 12.9% (22/170) in the low-risk group (*P* < 0.001), representing a six-fold increase in risk. Notably, the high-risk group accounted for 60.7% of all PPC events while comprising only one-fifth of the study population. These findings provide a research foundation for developing bedside risk assessment tools or integrating them into clinical decision support systems, and suggest that high-risk children may benefit from intensified perioperative monitoring and early preventive interventions.

The clinical significance of this study lies in identifying a set of easily obtainable objective laboratory indicators that can be used to identify children at high risk for PPCs preoperatively and in the early postoperative period. By identifying the inflection point for PPCs occurrence in children with elevated preoperative CRP, we can implement targeted measures including nutritional support, management of underlying infections or inflammatory conditions (e.g., use of antibiotics, deferring elective surgery), and preoperative respiratory physiotherapy to improve baseline physiological status and reduce PPCs incidence. Intraoperative anti-inflammatory strategies, including administration of glucocorticoids, ulinastatin, or dexmedetomidine, can suppress postoperative CRP elevation and potentially reduce the incidence of pulmonary complications (20–22). Our findings also suggest that for patients with abnormal CRP and albumin levels on postoperative day 1, enhanced monitoring and intervention beyond routine care should be implemented, including serial laboratory assessments, intensified respiratory physiotherapy (e.g., lung recruitment maneuvers and active airway clearance techniques), and individualized fluid management guided by goal-directed therapy strategies to avoid fluid overload or hypovolemia. Furthermore, continuous monitoring of pulse oximetry and end-tidal carbon dioxide in the post-anesthesia care unit or pediatric intensive care unit may facilitate early warning and timely intervention for pulmonary complications. Collectively, these measures enable a proactive, individually tailored perioperative management strategy, with the potential to reduce both the incidence and severity of PPCs in the pediatric population.

This study has several limitations. First, as a single-center retrospective study, selection bias and information bias are inevitable. Second, the exclusion of patients who required postoperative transfer to the intensive care unit (ICU) or mechanical ventilation may have introduced selection bias, potentially leading to an underestimation of the true incidence of postoperative pulmonary complications (PPCs). Therefore, our findings may not be generalizable to this higher-risk patient population. Third, although the sample size (*n* = 214) was sufficient for preliminary modeling, the model requires external validation in larger, multicenter prospective cohorts to confirm its generalizability. Fourth, although image-defined risk factors (IDRFs) are recognized as critical for preoperative assessment in neuroblastoma (23), they were not included in our final prediction model because they were not independently associated with PPCs in multivariate analysis (*P* > 0.05). This lack of association may be explained by the high proportion of patients who received preoperative chemotherapy (63.1%) in our cohort, which could have altered the baseline characteristics of IDRFs. Indeed, we found that a history of preoperative chemotherapy was associated with an increased risk of PPCs, possibly because certain IDRFs show limited response to chemotherapy and may persist as perioperative risk factors (24). Future prospective studies with standardized IDRF assessment are needed to clarify their role in predicting PPCs in children undergoing resection of abdominal neuroblastoma. Fifth, while we adjusted for multiple known confounders, unmeasured confounding variables (e.g., details of specific anesthetic agents, differences in postoperative analgesia protocols) may still influence the results.

## 5 Conclusion

This study demonstrates that preoperative CRP levels and early postoperative CRP and albumin levels are independent risk factors for PPCs in children undergoing neuroblastoma resection. The prediction model based on these indicators demonstrates excellent predictive performance and holds promise for assisting clinicians in individualized risk assessment and intervention, thereby potentially improving patient outcomes. Future prospective studies are needed to further validate and optimize this model.

## Data Availability

The original contributions presented in the study are included in the article/Supplementary Material, further inquiries can be directed to the corresponding authors.
