# Supplementary material for: Incidence and risk factors for postoperative pulmonary complications in children following surgery for retroperitoneal neuroblastoma
Source: Front Pediatr. 2026 May 19;14:1817205. doi: 10.3389/fped.2026.1817205 (PMC13226564; doi:10.3389/fped.2026.1817205)
Supplement: Supplementary file 1 [file Table1.docx]

**TABLE S1.** Results of Multivariate Logistic Regression Analysis for Predictive Factors of Postoperative Pulmonary Complications

| **Variables** | **B** | **S.E.** | **wald** | **p-value** | **aOR** | **(95%CI)** | |
| --- | --- | --- | --- | --- | --- | --- | --- |
|  |  |  |  |  |  | **LOWER** | **UPPER** |
| BMI | -0.187 | 0.155 | 1.441 | 0.230 | 0.830 | 0.612 | 1.125 |
| Operative time | 0.272 | 0.163 | 2.788 | 0.095 | 1.312 | 0.954 | 1.805 |
| Maximum diameter of tumour at surgery | -0.012 | 0.112 | 0.011 | 0.916 | 0.988 | 0.793 | 1.231 |
| Number of resected lymphatic regions | 0.175 | 0.137 | 1.633 | 0.201 | 1.191 | 0.911 | 1.558 |
| Volume of infused crystalloids | 0.056 | 0.078 | 0.523 | 0.469 | 1.058 | 0.908 | 1.232 |
| Estimated blood loss | 0.012 | 0.007 | 2.958 | 0.085 | 1.012 | 0.998 | 1.026 |
| Preoperative haemoglobin | -0.007 | 0.026 | 0.073 | 0.787 | 0.993 | 0.944 | 1.045 |
| Preoperative C-reactive protein | 0.305 | 0.099 | 9.464 | 0.002 | 1.357 | 1.117 | 1.648 |
| Preoperative creatinine | -0.001 | 0.073 | 0.000 | 0.987 | 0.999 | 0.866 | 1.152 |
| C-reactive protein on Postoperative day | 0.036 | 0.017 | 4.607 | 0.032 | 1.037 | 1.003 | 1.072 |
| Albumin on Postoperative day | 0.238 | 0.079 | 8.968 | 0.003 | 1.268 | 1.086 | 1.482 |
| Pathology | 0.526 | 0.652 | 0.652 | 0.419 | 1.693 | 0.472 | 6.073 |
| International Neuroblastoma Risk Group stage | -1.727 | 0.911 | 3.592 | 0.058 | 0.178 | 0.030 | 1.061 |
| International Neuroblastoma Risk Group risk stratification | 1.618 | 0.956 | 2.869 | 0.090 | 5.045 | 0.775 | 32.829 |
| Preoperative chemotherapy history | 1.085 | 1.097 | 0.979 | 0.322 | 2.960 | 0.345 | 25.390 |
